# Assessing Seed Longevity of the Invasive Weed Navua Sedge (*Cyperus aromaticus*), by Artificial Ageing

**DOI:** 10.3390/plants11243469

**Published:** 2022-12-11

**Authors:** Aakansha Chadha, Singarayer K. Florentine, Kunjithapatham Dhileepan, Christopher Turville

**Affiliations:** 1 Future Regions Research Centre, Federation University Australia, Mount Helen, VIC 3350, Australia; 2 Applied Chemistry and Environmental Science, School of Science, STEM College, RMIT University, 124 La Trobe St., Melbourne, VIC 3000, Australia; 3 Department of Agriculture and Fisheries, Biosecurity Queensland, Ecosciences Precinct, Dutton Park, QLD 4102, Australia; 4 Institute of Innovation, Science and Sustainability, Federation University Australia, Mount Helen, VIC 3350, Australia

**Keywords:** seed longevity, artificial ageing, perennial weed, sedge, seed ageing

## Abstract

Navua sedge (*Cyperus aromaticus* (Ridley) Mattf. & Kukenth) is a significant agricultural and environmental weed found in tropical island countries including north Queensland, Australia. It is a prolific seed producer and consequently forms a high-density seedbank, and therefore understanding the longevity and persistence of the seeds can provide critical information required for the management of this species. A laboratory-controlled artificial ageing experiment was conducted where the seeds were exposed to a temperature of 45 °C and 60% relative humidity for 125 days. Seeds were removed at various times (1, 2, 5, 9, 20, 30, 50, 75, 100 and 125 days) and their viability determined through standard germination tests. It took 20 days in the artificial ageing environment for the seeds to decline to 50% viability which indicates that Navua sedge has relatively short-lived persistent seeds. These findings will assist in developing a better understanding of the seedbank dynamics of this invasive species, allowing managers to tactically implement control strategies and prepare budgets for ongoing treatments, and have implications for the duration and success of management programs.

## 1. Introduction

Navua sedge (*Cyperus aromaticus* (Ridley) Mattf. & Kukenth), is an invasive perennial C_4_ sedge of tropical environments which forms dense stands, has prolific seed production and has a creeping rhizome. This aggressive weed is a major cause of concern for the agricultural industry, causing productivity losses in pastures, which affects milk and livestock industries, and also in crops such as rice, sugarcane and banana [1,2,3]. More than 500 beef producers, dairy farmers and hay producers in the Atherton Tableland region of tropical north Queensland, Australia are affected by this weed. It is a major weed in Fiji where it has been known to reduce the carrying capacity of the pastures by up to 40%, thus reducing milk production [4,5]. It also causes related environmental problems and is seen growing along roadsides and creeks, in ditches, drains and lawns.

Navua sedge is an extremely aggressive persistent weed, which competes with pasture species and crops for light, water, nutrients and space and has the ability to quickly smother competing plants [6]. The plant reproduces both by seed and vegetatively [2,6], making it a very successful colonizer. Vegetatively, it spreads through the natural extension of the rhizome system and when viable rhizome fragments are dispersed during cultivation. It also spreads via seeds where each seed head produces approximately 250 seeds, and a dense stand of this weed (200 plants/m^2^) may produce viable seeds ranging from 44,400 to 56,700 seeds/m^2^ [6]. The seeds are light in weight and are readily dispersed over short distances when released in winds. However, the main vectors of seed dispersal are cattle, birds, humans, flood waters and harvesting machinery [2]. 

Management efforts are often focused to reduce the above-ground cover of weeds while efforts to manage their seedbank are not given comparable importance. However, recent study has shown the presence of more than 35,000 viable Navua sedge seeds/m^2^ in the soil seedbank in grazing pastures of tropical north Queensland [7] which is clearly of concern. This high density of seeds in the soil seedbank will contribute to the maintenance of future Navua sedge populations, but it is noted that these seeds have a finite lifespan and variable periods over which their seedbank remains viable. The current categorisation of seedbank resilience refers to (i) transient seedbanks, which do not persist in the soil for more than one-year, (ii) short-term persistent seedbanks, which can remain viable from one to five years, and (iii) long-term persistent seedbanks, that are viable for at least five years [8]. Seed deterioration is regulated by complex interactions between internal genetically controlled factors and external environmental factors [9]. The internal factors for seed persistence are possibly associated with DNA repair and transcription, the amount of reactive oxygen species produced, embryonic development and aspects of sugar metabolism [10,11]. Hence, seedbank persistence can vary between species, biotypes, different environmental factors and disturbances experienced by the seeds [12,13]. 

Two most used methods for testing seed longevity are seed burial in the field (in situ) and artificial ageing (ex situ). Studies on seed longevity in the field provide accurate information since they naturally involve both biotic and abiotic elements. These investigations, however, are not always feasible due to lengthy time requirements and considerable financial resource demands [14]. By comparison, artificial seed ageing under controlled settings is a valuable ex situ tool for predicting the potential of weed seeds to persist in field conditions [15]. Accelerated seed ageing can be achieved artificially by exposing the seeds to high relative humidity and high temperature conditions which helps to predict germination response of a particular weed species over a long- time period, in a relatively short span of time [16]. A crucial step in the management of soil seedbank is to determine the timeline and cost for management measures for which knowledge of the seed longevity is required. Until recently, no such study has been conducted on Navua sedge. Therefore, the objective of this study was to evaluate the lifespan of Navua sedge seeds by using an accelerated seed ageing method.

## 2. Results

**Figure 1:**
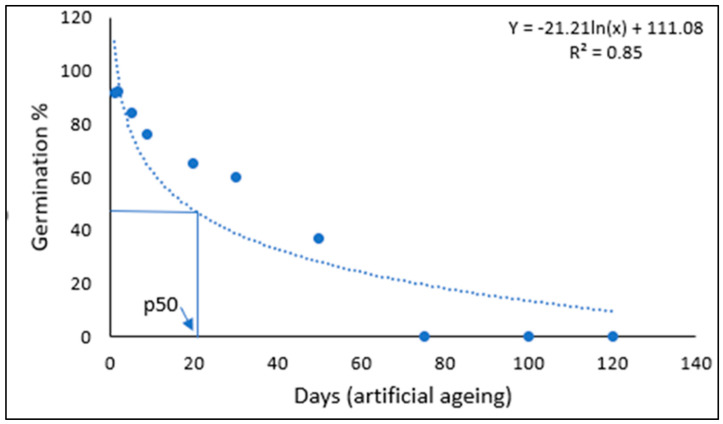
Logistic regression analysis of artificial ageing of Navua sedge seeds showing the time taken for seed viability to fall to 50% (P_50_) value.

The viability of seeds declined with time following a negative regression curve, with no germination at day 75 and beyond (Table 1, Figure 1). According to the fitted regression curve, the time taken for seed viability to fall to 50% (P_50_) was 20 days (Figure 1). The effect of artificial ageing on Navua sedge seeds showed no significant difference in the germination (%) between Days 0, 1 and 2, or Days 20 and 30, but a significant difference (*p* < 0.05) was observed between these two groups and other ageing times. Mean germination time also increased with the number of artificial ageing days (Table 1). Seeds removed at Day 50 had the highest mean germination time of 16.8 days compared to 10 days for seeds removed at day 1 and 2.

## 3. Discussion

Artificial ageing literature suggests species with P_50_ values between 20 days and 50 days generally have short-lived persistent seedbanks [14]. The regression equation fitted to the artificial ageing data suggests that approximately 50% of the seeds would maintain viability after 20 days of ageing and approximately 30% of the seeds would maintain viability after 50 days of exposure to the ageing environment. Result obtained in the artificial ageing experiment is moderately consistent with the findings of seedbank depletion studies conducted on Navua sedge which had recorded 31% seed viability after five years [6]. In contrast, recent study on Navua sedge seeds artificial ageing experiment found this species seeds to be long-lived with a P_50_ value of 82 days [17]. What is not clear from this study is the age of the seeds used for this trial, and the condition in which the seeds were stored. Further studies involving multiple populations and seeds of various ages are warranted based on this inconsistency. Some guidelines for the management of Navua sedge seedbank can be informed based on the findings of this study, combined with that of seedbank depletion studies [6], soil seedbank analysis [7] and seed germination requirements of Navua sedge [18]. As Navua sedge seeds require light for germination and do not emerge when buried deeper than 2 cm, the soil seedbank can potentially be manipulated through tillage at certain intensities [18]. The weed seedbank can reduce in abundance by encouraging germination of buried seeds via surface tillage that tend to bring Navua sedge seeds to the surface, or by using more intense tillage that can bury the seeds deep enough to prevent them from emerging. However, the persistent nature of the seeds should be considered when burying the seeds deeper than 2 cm as more than 30% seeds remain viable after 5 years [6].

Seed longevity as determined by artificial ageing can differ with in situ seed longevity. Observed persistence in the field is influenced by multiple factors, including the microhabitat of the seed as determined by the position of the seed within the seedbank [13]. Reduced seed viability is observed in seeds with shallow burial depth and increased duration of burial compared to seeds with deeper burial [19,20]. Seed predation is another factor which affects seed persistence in the soil, especially in the shallow soil layers [21]. Seed dormancy is usually prolonged at increased burial depths, especially for photoblastic seeds like Navua sedge [18,22]. In terms of deeper understanding of seedbank properties, future work should incorporate in situ seed burial trials to identify how both biotic and abiotic factors such as burial depth, soil moisture, soil temperature, predators and pathogens influence seed longevity. 

Our results observed significant increase in the mean germination time from day 20 onwards and decrease in germination index from day 5 onwards for seeds exposed to the ageing conditions. An increase in the mean germination time and decline in the germination index are one of the first indicators of seed ageing [23,24,25]. The mean germination time increases as the seeds have to recover and repair the damage processes prior to germination. Detrimental effects of seed ageing has been observed on the seed’s morphology, physiology and biochemical processes [26]. It has been suggested that suitable hydration can assist the seeds to recover from the damaging effects of the ageing process and could possibly extend the seed’s longevity [24,25]. Higher mean germination times have been shown to reduce the elongation rate and mean emergence time of subsequent seedlings [23,24]. Hence, future work should also consider evaluating the competitiveness of aged seeds of Navua sedge. 

Management implications derived from the artificial ageing treatments are limited as these studies do not consider environmental variables or spatial and temporal differences of a population that can prolong or break seed dormancy. However, the knowledge gained from this study about Navua sedge seed longevity can assist in understanding its invasive potential, future monitoring and in seedbank management programs. Immediate and short-term control and management efforts for Navua sedge should focus upon interruption of seed production, but long-term efforts must target seedbank management.

## 4. Materials and Methods

### 4.1. Seed Collection 

Mature seeds were collected from over 50 Navua sedge plants in early March 2020 along the roadside which had a monoculture of plants in South Johnstone, Queensland, Australia (17°42′55″ S 146°2′42″ E). In the field seeds were placed in labelled paper bags and transported to Federation University, Mount Helen campus, Victoria, Seed Ecology lab and stored in a dark cupboard until commencement of the experiment. The experiment was carried out between September 2021 and March 2022 at Mount Helen campus of Federation University Australia.

### 4.2. Preparation 

Seed bags for artificial ageing were prepared by placing twenty mature Navua sedge seeds into 2 cm × 2 cm square bags made of fine (0.05 mm) nylon mesh, which were sealed using hot glue. A total of 72 seed bags were used in the experiment, out of which six bags were used to test for rehydration and 66 bags for the artificial ageing.

### 4.3. Seed Rehydration

Protocol for comparative seed longevity testing developed by the Millennium Seed Bank Project, Royal Botanical Gardens, Kew, United Kingdom was used in this study for artificial ageing of the seeds [27]. For the rehydration process, the seed bags were placed on a stand above the rehydration solution (prepared by adding 385 g lithium chloride to 1 L water) at 47% relative humidity (RH) within a sealed electrical enclosure box. This box was then placed in an incubation chamber (Thermoline Scientific and Humidity cabinet, TRISLH-495-1SD, Vol.240, Sydney, Australia), maintained at 20 °C in darkness for 14 days. Following rehydration, seeds from six bags were used to measure the equilibrium RH (eRH) of the seeds using a hygrometer (HygroPalm HP23-A/HP23-AW-A handheld indicator, Rotronic AG, Bassersdorf, Switzerland). 

### 4.4. Seed Ageing

Following the rehydration step, the 66 selected seed bags were moved to a second electrical enclosure box with the ageing solution (prepared by adding 300 g lithium chloride to 1 L water) at 60% RH. This box was then placed in the incubation chamber maintained at 45 °C in darkness for 125 days. Six replicate bags were randomly selected and removed at each of the following ageing times: 0, 1, 2, 5, 10, 20, 30, 50, 75, 100 and 125 days. 

### 4.5. Germination Test

The seeds removed at each removal time were tested for viability using a standard germination test. Seeds from each bag were placed on separate 9 cm Petri dishes lined with sterilised Whatman^®^ No 10-filter paper and moistened with sterilised RO water to provide adequate moisture for germination and sealed with parafilm to ensure moisture retention. The Petri dishes were then placed in the incubation chamber equipped with cool-white fluorescent lamps to provide a photosynthetic photon-flux of 40 µmol m^−2^ s^−1^, maintained at 25/15 °C day/night temperature and 12 h light/12 h darkness [18]. The observations were made on alternate days for 35 days and seeds were regarded as germinated when the radicle was approximately 2 mm long. On the 35th day, all the non-germinated seeds were assessed for their viability, using 2,3,5-triphenyltetrazolium chloride (TTC) [28,29]. 

### 4.6. Analysis

The data was Log_10_ transformed and a Probit analysis performed in SPSS statistical software to find the P_50_ value, which is the time for viability to decline by 50% [27]. Seed viability was calculated for each removal time by averaging the germination of all the six replicates. The seed viability (germination %) was then plotted against time (number of artificial ageing days) to create a seed survival curve. Subsequently, a logistic regression model was fitted to the data and a Probit analysis was used to fit the viability equation.

Probit = 2.224 − 1.730 log_10_ No of days (1)

Mean germination time, which expresses the rate of seed germination, was calculated with the formula described by Kader [30]:

(2) $$MGT=\frac{\sum Dn}{\sum n}$$

where *n* represents number of seeds germinated on day *D*, and *D* is the number of days counted since the beginning of the germination trial.

A second formula given by Kader [29] was used to calculate the germination rate index (GI) to measure the rate of germination of Navua sedge seeds:

(3) $$GI=\frac{G1}{1}+\frac{G2}{2}+\ldots \ldots +\frac{Gx}{x}$$

where *G*1, *G*2 and *Gx* are the germination percentages × 100 at 1, 2 and x days after sowing, respectively.

## Figures and Tables

**Table 1:** Mean germination %, mean germination time (days) and germination index (±standard error) of artificially aged seeds of Navua sedge removed at various ageing times.

Ageing Times	Mean Germination %	Mean Germination Time (days)	Germination Index
Day 0	93.3 ± 2. 11	12.9 ± 0.66	1.6 ± 0.11
Day 1	91.7 ± 1.67	10.4 ± 0.31	1.9 ± 0.07
Day 2	92.5 ± 2.14	10.5 ± 0.48	1.8 ± 0.07
Day 5	84.2 ± 2.01	12.9 ± 0.84	1.4 ± 0.1
Day 9	75.8 ± 3.01	12.9 ± 0.33	1.2 ± 0.06
Day 20	65.0 ± 2.24	14.2 ± 0.69	1.0 ± 0.06
Day 30	60.0 ± 4.47	14.6 ± 0.45	0.9 ± 0.07
Day 50	36.7 ± 6.67	16.8 ± 0.56	0.5 ± 0.09
Day 75	0.0 ± 0.0	NA	NA
Day 100	0.0 ± 0.0	NA	NA
Day 125	0.0 ± 0.0	NA	NA
